# Survey dataset on occupants׳ satisfaction with housing and life in public residential estates in Ogun State, Nigeria

**DOI:** 10.1016/j.dib.2018.09.036

**Published:** 2018-09-15

**Authors:** Eziyi O. Ibem, Egidario B. Aduwo, Babalola O. Daniel, Emmanuel A. Ayo-Vaughan, Michael N. Odoanyanwu

**Affiliations:** a Department of Architecture, Covenant University, Ota, Ogun State, Nigeria; b Department of Architecture, Bells University of Technology, Ota, Ogun State, Nigeria; c Department of Architecture, Nnamdi Azikiwe University, Awka, Anambra State, Nigeria

**Keywords:** Residential satisfaction, Satisfaction with life, Government housing estate, Urban areas, Survey

## Abstract

This article describes the data associated with the research paper entitled “Subjective life satisfaction in public housing in urban areas of Ogun State” by Ibem and Amole (2013). A multistage sampling technique and questionnaire were used to extract data from occupants in 517 dwelling units in ten selected government mass housing schemes located in five urban centers in Ogun State, Nigeria. The dataset has 57 different variables describing households’ demographics, dwelling units’ features, supply of basic services, and neighbourhood environment in the residential estates. In addition, it also contains variables used to assess residential satisfaction and satisfaction with life. The Statistical Package of the Social Sciences (SPSS) file, descriptive statistics for all the variables and questionnaire used to derive the data are provided in this article. The dataset will enhance understanding of the main research findings and conclusions.

**Specifications table**

**Table:** 

Subject area	Architecture
More specific subject area	Housing
Type of data	Tables and Figures
How data was acquired	Field Survey
Data format	Analysed
Experimental factors	Multi-stage sampling technique was used in the selection of residents in 10 government constructed mass housing projects
Experimental features	The dataset was derived from household heads or their representatives in 10 government residential estates in five urban centers in Ogun State
Data source location	Ogun State, Nigeria
Data accessibility	The data are accessible in this paper

**Value of the data**

• The dataset is valuable as it provides insight into the housing situations of occupants in government mass housing projects in five urban centers of Ogun State southwest, Nigeria.
• The data also have value in understanding the standard of living of occupants in selected mass housing schemes developed by the government in Ogun State.
• Housing researchers can use the dataset to compare the housing situations of occupants in other government residential mass housing estates and types of urban housing environment.
• The dataset can be used to assess the performance of government constructed mass housing schemes in meeting the needs and expectations of end-users.
• The data and questionnaire instrument can be valuable in similar studies in the future.

## 1 Data

The dataset described in this article was extracted from 517 residents in the (i) Workers Housing Estate, Laderin (ii) Ogun State Housing Corporation Estate, Ajebo (iii) Obasanjo Hill-Top (GRA) Estate (iv) Kemta Extension Housing Estate, Olokota (v) Media Village, Ajebo; and (vi) Otunba Gbenga Daniel Housing Estate, Asero in Abeokuta. Others are the Ogun State Housing Corporation, Idiroko Road, Ota; Otunba Gbenga Daniel-Sparklight housing estate, Ibafo; Ogun State Property Investment Corporation (OPIC) Estate, Agbara and Otunba Gbenga Daniel Housing Estate, Itanrin, Ijebu-Ode in Ogun State, southwest Nigeria [1], [2], [3].

The dataset comprises nominal, interval and ordinal data and contains nine demographics variables (sex, marital status, age, education, employment status, income, length of stay in the residence, household size, tenure status) and 16 objective housing characteristics, including the number of bedrooms, type of residence, location and use of kitchen, type of walling and ceiling materials, window and door types, wall and floor finishes, type of roofing materials, mode of acquisition of residence and state of repair of the buildings, and modes of water supply, main source of power supply, domestics waste disposal arrangement. It also has 31 subjective variables drawn from dwelling units’ characteristics, sources of water and electricity in the dwelling units; physical, social and economic characteristics of the neighbourhood environments as well as management practices in the residential estates, used to investigate residential satisfaction [1], [2], [4] and one subjective variable used to assess residents’ satisfaction with life in the estates [5].

Detailed descriptions of each of these variables can be found in the sample copy of questionnaire instrument, the SPSS file, and residents’ responses to the questionnaire included in the supplementary files and made available in this article.

## 2 Experimental design, materials, and methods

The dataset was sourced from ten mass housing estates constructed between 2003 and 2010 by the Government of Ogun State using the Core housing, Shell, Public-Private Partnerships (PPPs) and Turnkey strategies in Abeokuta, Ota, Ijebu- Ode, Ifo and Agbara. Based on the research by Ibem and Amole [1], a questionnaire was designed for the survey. The use of questionnaire as a survey instrument can be seen in similar works by previous authors [6], [7], [8], [9], [10]. The questionnaire had mainly closed-ended questions and three sections: A, B and C. Section A was used to gather data on nine variables describing the socio-economic and demographic profiles of the housing occupants. Section B had questions on 16 housing characteristics, while Section C dwelt on residents’ levels of satisfaction with 31 components of their home environment (i.e. residential satisfaction) and one question on satisfaction with life in the estates (see the Supplementary file). In collecting data on residents’ levels of satisfaction with their housing situations and satisfaction with life in the residential estates, a 5-Point Likert type scale starting from ‘1’ for *Very Dissatisfied* and ending with ‘5’ for *Very Satisfied* was used. Similar studies have used this scale of measurement as seen in the previous works by [8], [9], [10], [11].

A cross-sectional survey involving the administration of pre-tested questionnaire was used to gather the data from residents in the selected estates. This is in line with the approach adopted by previous authors in similar studies [12], [13], [14]. Of the 1411 housing units of different typologies constructed in the ten housing estates listed earlier on, 709 dwelling units were occupied at the time of conducting the survey [1], [3]. Consequently, a multistage sampling technique was adopted in selecting the number of dwelling units included the survey. Whereas census method of selection was used for houses developed via the Shell and PPPs strategies due to their relatively low number in the total population of housing units occupied by residents, quota sampling technique was used to select the number of houses developed using the Core housing and Turnkey strategies included in the survey. This resulted to a sample size of 670 housing units/ households for the survey. Similar approach has been used in previous studies [1], [2], [3], [4].

In the survey, each of the 15 and 30 housing units occupied in the residential estates developed through Shell and PPPs strategies, respectively, was given a questionnaire to fill, however, while in the residential estates developed using the Core housing and Turnkey strategies, random sampling method was applied in selecting dwelling units for the administration of questionnaire to the occupants. The questionnaire was administered by hand to household heads or their representatives in each of the dwelling units selected in the morning and evening hours of week days and daytime at weekends between December 2009 and February 2010. Although 670 questionnaires were distributed in all the ten estates, 517 questionnaires retrieved were found to have been correctly filled by the respondents. The SPSS software package was used to analyse the data and the responses are presented using tables and figures as shown in the supplementary files.
