# Supplementary material for: Survey dataset on occupants׳ satisfaction with housing and life in public residential estates in Ogun State, Nigeria
Source: Data Brief. 2018 Sep 15;20:1745–8. doi: 10.1016/j.dib.2018.09.036 (PMC6161412; doi:10.1016/j.dib.2018.09.036)
Supplement: Supplementary file 2 — Supplementary material [file mmc2.zip › Sample of Questionniare .doc]

Dear Respondent,

This questionnaire is designed to elicit responses on issues relating to the public housing programmes in Ogun State between 2003 and 2010. It is mainly an instrument for gathering data for an on-going research on public housing. All information provided will be treated confidentially, and used purely for academic purpose.

Thanks for providing responses to the questions

**INSTRUCTION**: Please **tick (√) or fill** as appropriate

SECTION A: **BASIC INFORMATION**

Name of Housing Estate-------------------------------------------------------------------------------------------------

1. Sex	(i) Male [ ]	(ii) Female [ ]

2. How old are you? (i) 18-30years [ ] (ii) 31-45 years [	] (iii) 46-59years[	] (iv) 60years and above [	]

3. Marital status: (i) Single [ ]	(ii) Divorced [ ]	(iii) Married [ ]	(iv) Widowed [ ]

4. What is the highest level of your educational attainment? (i) First School Leaving Certificate [ ]

(ii) WASC O’Level [ ] (iii) OND [ ] (iv) NCE[ ] (v) HND [ ] (vi) Bachelor Degree [ ] (vii) Masters

degree [	] (viii) Ph.D [	] (ix) Others----------------------------------------------------------------------------

5. What is your employment sector (i) Government [	] (ii) Private organisation [	] (iii)

Self-employed [	] (iv) Retiree [	] (v) Others--------------------------------------------------------------------

6. The range of your average monthly income is? (i) Below N 13, 000 [ ] (ii) N14, 000- N 37,000 [ ]

(iii) N 38,000- N 44,000 [ ] (iv) N45- N 71,000 [ ]	(v) N 72,000- N 145,000[ ]

(vi) Above N 146,000[	]

7. How long have you been living in this house? (i) Less than 1year [ ] (ii) 1-3years [	]

(iii) 4-5 years [	]	(iv) 5 years+[	]

8. How many persons are in your household? (i) 1 [ ] (ii) 2 [ ](iii) 3 [ ]	(iv) 4 [	] (v) Above 4 [	].

9. What is the type of tenure of the house you are occupying? (i) Privately rented[	]

(ii) Family house [	] (iii) Owner occupied [	]	(iv) Official quarters[ ] (v) Free Occupation [	]

(vi) Others, please specify--------------------------------------------------------------------------------------------

SECTION B: **HOUSING CHARACTERISTICS**

1. How many bed rooms do you have in your apartment? (i) 1 [ ] (ii) 2 [	]	(iii) 3 [	] (iv) 4 [ ]

(v) 5 bed rooms+ [	]

2. Where is your kitchen located? (i) Within the building [ ] (ii) Detached from the building [ ]

(iii) There is no kitchen for the building [	]

3. How is the use of kitchen? (i) Exclusively used by your family [	]	(ii) Shared with other families [	]

4. What is the mode of acquisition of residence? (i) Bought directly from the developers [ ] (ii) Bought from previous owner (s) [ ] (iii) Government allocation[ ]	(iv) By inheritance [ ] (iv) Mortgage arrangement [ ] (v) Gift [	]	(vi) Others, please specify----------------------------------------------------------

5. What is the predominant source of water supply in your apartment? (i) Water vendors [ ] (ii) Wells outside the building [	] (iii) Borehole within the estate [ ] (iv) Public water supply system [ ]	(v) Others, Please specify-----------------------------------------------------------------------------------------------------

6. What is the main source of power supply in your apartment? (i) Personal Power Generating sets [ ]

(ii) Power Generating Plant in the estate [ ] (iii) Solar Panels [	](iii) Public Power supply[	]

(iv) None of the above [ ] (v) others, please specify----------------------------------------------------------------

7. Who is responsible for domestics disposal in this housing estate? (i) Residents [ ] (ii) Contractors [ ] (iii)	Estate Managers[	] (iv) Government agencies[ ]

8. Housing Typology (i) Single-Family Bungalow [	]	(ii) Semi detached Bungalow [	]

(iii) Detached stored [	] (iv) Semi-detached stored building (Block of flats) [	]	(v) Duplex [	]

(vi) Others, please specify-----------------------------------------------------------------------------------------

9. Walling material of your house is (i) Sun dried burnt bricks [ ] (ii) Compressed Stabilized Laterite [	]

(iii) Sancerre Cement Blocks [ ] (iv) Others-------------------------------------------------------------------

10. Wall finishing (i) Cement sand plastering [	] (ii) Painted [	] (iii) Others-----------------------------------

11. Type of windows used in the house (i) Timber [ ] (ii) Glazed louvers [	] (iii) glazed aluminium [	]

12. The type doors used in the house (i) Plywood flushed [ ] (ii) Panelled timber [ ] (iii) Aluminium

Glazed [	] (iv) Panelled Steel [	] (v) others---------------------------------------------------------------

13. Type of floor finishes in your house (i) Cement screed [	] (ii) PVC Tiles [	] (iii) Ceramic Tiles [	]

(iv) Terrazzo [	] (v) Marble [	] (vi) Others, please specify--------------------------------------------------

14. Ceiling Material(s) (i) Asbestos [	]	(ii) Mineral Fibre [	]	(iii) Acoustic ceiling [	]	(iv) PVC strips [	] (v) Polished timber [	] (vi) Plaster of Plaster (POP) [	]

15. Roofing material used in your house is (i) Galvanized iron [	] (ii) Asbestos [	] (iii) Aluminium long span [ ] (iv) Villa tiles [	] (v) others, please specify----------------------------------------------------------

16. State of repair of the building? (i) Dilapidated [	] (ii) Major repairs [	] (iii) Minor repairs [	]

(iv) Sound [	]

**SECTION C: SATISFACTION WITH HOUSING & SATISFACTION WITH LIFE IN THE**

**RESIDENCE**

**How satisfied or dissatisfied are you with the house and housing estate where you live in terms of the following? Please** tick (√)

| S/N | Attributes | Very  Dissatisfied | Dissatisfied | Fair | Satisfied | Very  Dissatisfied |
| --- | --- | --- | --- | --- | --- | --- |
| 1 | Sizes of Living & Dining spaces |  |  |  |  |  |
| 2 | Sizes of bedrooms |  |  |  |  |  |
| 3 | Number of bed rooms |  |  |  |  |  |
| 4 | Sizes of cooking and storage spaces |  |  |  |  |  |
| 5 | Type of house you live in |  |  |  |  |  |
| 6 | Bathroom/Toilets facilities in your house |  |  |  |  |  |
| 7 | Types of building materials used in your house |  |  |  |  |  |
| 8 | Location of your apartment |  |  |  |  |  |
| 9 | External appearance of the house you live in |  |  |  |  |  |
| 10 | Natural lighting and circulation of fresh air in your living and bed rooms |  |  |  |  |  |
| 11 | Noise level in your house and the housing estate |  |  |  |  |  |
| 12 | Water supply and sanitary services in your house |  |  |  |  |  |
| 13 | Electrical services in your house |  |  |  |  |  |
| 14 | Level of privacy in your house |  |  |  |  |  |
| 15 | Cost of acquiring/ rentage of your house |  |  |  |  |  |
| 16 | Nearness of your house to recreational/sport facilities |  |  |  |  |  |
| 17 | Nearness of the housing estate to public infrastructure and urban services |  |  |  |  |  |
| 18 | Nearness to shopping facilities within the estate |  |  |  |  |  |
| 19 | Nearness of your house to place of work |  |  |  |  |  |
| 20 | Nearness of your house to health care facilities |  |  |  |  |  |
| 21 | Nearness of your house to your children’s school |  |  |  |  |  |
| 22 | Nearness of your house to the nearest market |  |  |  |  |  |
| 23 | Prices of goods and services in the housing estate |  |  |  |  |  |
| 24 | Job/ business opportunities within and around the housing estate |  |  |  |  |  |
| 25 | Level of crime and anti- social activities in the housing estate where you live |  |  |  |  |  |
| 26 | Communal activities in your housing estate |  |  |  |  |  |
| 27 | Suitability of where you live to your natural way of life |  |  |  |  |  |
| 28 | Rules and regulations regarding residency in the housing estate you live in |  |  |  |  |  |
| 29 | Management and Maintenance of facilities in the housing estate |  |  |  |  |  |
| 30 | General cleanliness of the housing estate |  |  |  |  |  |
| 31 | General security of life and property in the estate |  |  |  |  |  |
| 32 | General level of satisfaction with life in the estate |  |  |  |  |  |
