# Supplementary material for: Survey dataset on occupants׳ satisfaction with housing and life in public residential estates in Ogun State, Nigeria
Source: Data Brief. 2018 Sep 15;20:1745–8. doi: 10.1016/j.dib.2018.09.036 (PMC6161412; doi:10.1016/j.dib.2018.09.036)
Supplement: Supplementary file 2 — Supplementary material [file mmc2.zip › Residents' Responses to the questions on residential sataifsction and Satisfaction with life .doc]

**Figure 1: Distribution of the residents according to levels of satisfaction with life in the housing estates**

**Figure 2: Distribution of the Residents according to satisfaction with their housing**

**situations in the estates**

**Residents’ Satisfaction with 31 Variables describing their residential environment**

| **Table 1: Satisfaction with the sizes of living/dining space** | | | | | |
| --- | --- | --- | --- | --- | --- |
|  | | Frequency | Percent | Valid Percent | Cumulative Percent |
| Valid | No Response | 2 | .4 | .4 | .4 |
|  | Very Dissatisfied | 8 | 1.5 | 1.5 | 1.9 |
|  | Dissatisfied | 27 | 5.2 | 5.2 | 7.2 |
|  | Undecided | 159 | 30.8 | 30.8 | 37.9 |
|  | Satisfied | 259 | 50.1 | 50.1 | 88.0 |
|  | Very Satisfied | 62 | 12.0 | 12.0 | 100.0 |
|  | Total | 517 | 100.0 | 100.0 |  |

| **Table 2: Satisfaction with size of bedrooms in the housing unit** | | | | | |
| --- | --- | --- | --- | --- | --- |
|  | | Frequency | Percent | Valid Percent | Cumulative Percent |
| Valid | No Response | 1 | .2 | .2 | .2 |
|  | Very Dissatisfies | 4 | .8 | .8 | 1.0 |
|  | Dissatisfied | 23 | 4.4 | 4.4 | 5.4 |
|  | Undecided | 117 | 22.6 | 22.6 | 28.0 |
|  | Satisfied | 299 | 57.8 | 57.8 | 85.9 |
|  | Very Satisfied | 73 | 14.1 | 14.1 | 100.0 |
|  | Total | 517 | 100.0 | 100.0 |  |

| **Table 3: Satisfaction with the number of bedrooms in the dwelling units** | | | | | |
| --- | --- | --- | --- | --- | --- |
|  | | Frequency | Percent | Valid Percent | Cumulative Percent |
| Valid | No Response | 3 | .6 | .6 | .6 |
|  | Very Dissatisfied | 10 | 1.9 | 1.9 | 2.5 |
|  | Dissatisfied | 204 | 39.5 | 39.5 | 42.0 |
|  | Undecided | 98 | 19.0 | 19.0 | 60.9 |
|  | Satisfied | 167 | 32.3 | 32.3 | 93.2 |
|  | Very Satisfied | 35 | 6.8 | 6.8 | 100.0 |
|  | Total | 517 | 100.0 | 100.0 |  |

| **Table 4: Satisfaction with the sizes of cooking and storage spaces** | | | | | |
| --- | --- | --- | --- | --- | --- |
|  | | Frequency | Percent | Valid Percent | Cumulative Percent |
| Valid | No Response | 3 | .6 | .6 | .6 |
|  | Very Dissatisfied | 12 | 2.3 | 2.3 | 2.9 |
|  | Dissatisfied | 96 | 18.6 | 18.6 | 21.5 |
|  | Undecided | 152 | 29.4 | 29.4 | 50.9 |
|  | Satisfied | 178 | 34.4 | 34.4 | 85.3 |
|  | Very Satisfied | 76 | 14.7 | 14.7 | 100.0 |
|  | Total | 517 | 100.0 | 100.0 |  |

| **Table 5: Satisfaction with the housing type** | | | | | |
| --- | --- | --- | --- | --- | --- |
|  | | Frequency | Percent | Valid Percent | Cumulative Percent |
| Valid | No Response | 4 | .8 | .8 | .8 |
|  | Very Dissatisfied | 5 | 1.0 | 1.0 | 1.7 |
|  | Dissatisfied | 30 | 5.8 | 5.8 | 7.5 |
|  | Undecided | 246 | 47.6 | 47.6 | 55.1 |
|  | Satisfied | 211 | 40.8 | 40.8 | 95.9 |
|  | Very Satisfied | 21 | 4.1 | 4.1 | 100.0 |
|  | Total | 517 | 100.0 | 100.0 |  |

| **Table 6: Satisfaction with bathroom and toilet facilities in the dwelling unit** | | | | | |
| --- | --- | --- | --- | --- | --- |
|  | | Frequency | Percent | Valid Percent | Cumulative Percent |
| Valid | No Response | 7 | 1.4 | 1.4 | 1.4 |
|  | Very Dissatisfied | 8 | 1.5 | 1.5 | 2.9 |
|  | Dissatisfied | 70 | 13.5 | 13.5 | 16.4 |
|  | Undecided | 226 | 43.7 | 43.7 | 60.2 |
|  | Satisfied | 188 | 36.4 | 36.4 | 96.5 |
|  | Very Satisfied | 18 | 3.5 | 3.5 | 100.0 |
|  | Total | 517 | 100.0 | 100.0 |  |

| **Table 7: Satisfaction with the location of housing unit within the estate** | | | | | |
| --- | --- | --- | --- | --- | --- |
|  | | Frequency | Percent | Valid Percent | Cumulative Percent |
| Valid | No Response | 4 | .8 | .8 | .8 |
|  | Very Dissatisfied | 1 | .2 | .2 | 1.0 |
|  | Dissatisfied | 45 | 8.7 | 8.7 | 9.7 |
|  | Undecided | 219 | 42.4 | 42.4 | 52.0 |
|  | Satisfied | 216 | 41.8 | 41.8 | 93.8 |
|  | Very Satisfied | 32 | 6.2 | 6.2 | 100.0 |
|  | Total | 517 | 100.0 | 100.0 |  |

| **Table 8: Satisfaction with natural lighting and ventilation of living and sleeping areas in the house** | | | | | |
| --- | --- | --- | --- | --- | --- |
|  | | Frequency | Percent | Valid Percent | Cumulative Percent |
| Valid | Very Dissatisfied | 2 | .4 | .4 | .4 |
|  | Dissatisfied | 27 | 5.2 | 5.2 | 5.6 |
|  | Undecided | 313 | 60.5 | 60.5 | 66.2 |
|  | Satisfied | 148 | 28.6 | 28.6 | 94.8 |
|  | Very Satisfied | 27 | 5.2 | 5.2 | 100.0 |
|  | Total | 517 | 100.0 | 100.0 |  |

| **Table 9: Satisfaction with the building materials used** | | | | | |
| --- | --- | --- | --- | --- | --- |
|  | | Frequency | Percent | Valid Percent | Cumulative Percent |
| Valid | Very Dissatisfied | 32 | 6.2 | 6.2 | 6.2 |
|  | Dissatisfied | 88 | 17.0 | 17.0 | 23.2 |
|  | Not Sure | 251 | 48.5 | 48.5 | 71.8 |
|  | Satisfied | 129 | 25.0 | 25.0 | 96.7 |
|  | Very Satisfied | 17 | 3.3 | 3.3 | 100.0 |
|  | Total | 517 | 100.0 | 100.0 |  |

| **Table 10: Satisfaction with the aesthetics of the house** | | | | | |
| --- | --- | --- | --- | --- | --- |
|  | | Frequency | Percent | Valid Percent | Cumulative Percent |
| Valid | No Response | 6 | 1.2 | 1.2 | 1.2 |
|  | Very Dissatisfied | 3 | .6 | .6 | 1.7 |
|  | Dissatisfied | 58 | 11.2 | 11.2 | 13.0 |
|  | Not Sure | 264 | 51.1 | 51.1 | 64.0 |
|  | Satisfied | 161 | 31.1 | 31.1 | 95.2 |
|  | Very Satisfied | 25 | 4.8 | 4.8 | 100.0 |
|  | Total | 517 | 100.0 | 100.0 |  |

| **Table 11: Satisfaction with the level of noise pollution within the estate** | | | | | |
| --- | --- | --- | --- | --- | --- |
|  | | Frequency | Percent | Valid Percent | Cumulative Percent |
| Valid | Very Dissatisfied | 9 | 1.7 | 1.7 | 1.7 |
|  | Dissatisfied | 19 | 3.7 | 3.7 | 5.4 |
|  | Not Sure | 265 | 51.3 | 51.3 | 56.7 |
|  | Satisfied | 179 | 34.6 | 34.6 | 91.3 |
|  | Very Satisfied | 45 | 8.7 | 8.7 | 100.0 |
|  | Total | 517 | 100.0 | 100.0 |  |

| **Table 12: Satisfaction with water supply in the dwelling unit** | | | | | |
| --- | --- | --- | --- | --- | --- |
|  | | Frequency | Percent | Valid Percent | Cumulative Percent |
| Valid | Very Dissatisfied | 43 | 8.3 | 8.3 | 8.3 |
|  | Dissatisfied | 289 | 55.9 | 55.9 | 64.2 |
|  | Undecided | 105 | 20.3 | 20.3 | 84.5 |
|  | Satisfied | 73 | 14.1 | 14.1 | 98.6 |
|  | Very Satisfied | 7 | 1.4 | 1.4 | 100.0 |
|  | Total | 517 | 100.0 | 100.0 |  |

| **Table 13: Satisfaction with power supply in the residence** | | | | | |
| --- | --- | --- | --- | --- | --- |
|  | | Frequency | Percent | Valid Percent | Cumulative Percent |
| Valid | No Response | 1 | .2 | .2 | .2 |
|  | Very Dissatisfied | 52 | 10.1 | 10.1 | 10.3 |
|  | Dissatisfied | 260 | 50.3 | 50.3 | 60.5 |
|  | Undecided | 128 | 24.8 | 24.8 | 85.3 |
|  | Satisfied | 65 | 12.6 | 12.6 | 97.9 |
|  | Very Dissatisfied | 11 | 2.1 | 2.1 | 100.0 |
|  | Total | 517 | 100.0 | 100.0 |  |

| **Table 14: Satisfaction with the level of privacy in the dwelling unit** | | | | | |
| --- | --- | --- | --- | --- | --- |
|  | | Frequency | Percent | Valid Percent | Cumulative Percent |
| Valid | No Response | 4 | .8 | .8 | .8 |
|  | Very Dissatisfied | 5 | 1.0 | 1.0 | 1.7 |
|  | Dissatisfied | 10 | 1.9 | 1.9 | 3.7 |
|  | Undecided | 104 | 20.1 | 20.1 | 23.8 |
|  | Satisfied | 294 | 56.9 | 56.9 | 80.7 |
|  | Very Satisfied | 100 | 19.3 | 19.3 | 100.0 |
|  | Total | 517 | 100.0 | 100.0 |  |

| **Table 15: Satisfaction with the cost of acquisition or rent paid for the residence** | | | | | |
| --- | --- | --- | --- | --- | --- |
|  | | Frequency | Percent | Valid Percent | Cumulative Percent |
| Valid | No Response | 11 | 2.1 | 2.1 | 2.1 |
|  | Very Dissatisfied | 4 | .8 | .8 | 2.9 |
|  | Dissatisfied | 38 | 7.4 | 7.4 | 10.3 |
|  | Undecided | 222 | 42.9 | 42.9 | 53.2 |
|  | Satisfied | 215 | 41.6 | 41.6 | 94.8 |
|  | Very Dissatisfied | 27 | 5.2 | 5.2 | 100.0 |
|  | Total | 517 | 100.0 | 100.0 |  |

| **Table 16: Satisfaction with the distance from your house to recreational facilities** | | | | | |
| --- | --- | --- | --- | --- | --- |
|  | | Frequency | Percent | Valid Percent | Cumulative Percent |
| Valid | No Response | 7 | 1.4 | 1.4 | 1.4 |
|  | Very Dissatisfied | 174 | 33.7 | 33.7 | 35.0 |
|  | Dissatisfied | 229 | 44.3 | 44.3 | 79.3 |
|  | Undecided | 78 | 15.1 | 15.1 | 94.4 |
|  | Satisfied | 28 | 5.4 | 5.4 | 99.8 |
|  | Very Satisfied | 1 | .2 | .2 | 100.0 |
|  | Total | 517 | 100.0 | 100.0 |  |

| **Table 17: Satisfaction with the distance to children's school from your house** | | | | | |
| --- | --- | --- | --- | --- | --- |
|  | | Frequency | Percent | Valid Percent | Cumulative Percent |
| Valid | No Response | 21 | 4.1 | 4.1 | 4.1 |
|  | Very Satisfied | 114 | 22.1 | 22.1 | 26.1 |
|  | Dissatisfied | 100 | 19.3 | 19.3 | 45.5 |
|  | Undecided | 206 | 39.8 | 39.8 | 85.3 |
|  | Satisfied | 68 | 13.2 | 13.2 | 98.5 |
|  | Very Satisfied | 8 | 1.5 | 1.5 | 100.0 |
|  | Total | 517 | 100.0 | 100.0 |  |

| **Table 18: Satisfaction with the distance from your house to the nearest market** | | | | | |
| --- | --- | --- | --- | --- | --- |
|  | | Frequency | Percent | Valid Percent | Cumulative Percent |
| Valid | No Response | 3 | .6 | .6 | .6 |
|  | Very Dissatisfied | 184 | 35.6 | 35.6 | 36.2 |
|  | Dissatisfied | 137 | 26.5 | 26.5 | 62.7 |
|  | Undecided | 140 | 27.1 | 27.1 | 89.7 |
|  | Satisfied | 45 | 8.7 | 8.7 | 98.5 |
|  | Very Satisfied | 8 | 1.5 | 1.5 | 100.0 |
|  | Total | 517 | 100.0 | 100.0 |  |

| **Table 19: Satisfaction with distance between your house and nearest shopping facilities** | | | | | |
| --- | --- | --- | --- | --- | --- |
|  | | Frequency | Percent | Valid Percent | Cumulative Percent |
| Valid | No Response | 8 | 1.5 | 1.5 | 1.5 |
|  | Very Dissatisfied | 211 | 40.8 | 40.8 | 42.4 |
|  | Dissatisfied | 181 | 35.0 | 35.0 | 77.4 |
|  | Not Sure | 83 | 16.1 | 16.1 | 93.4 |
|  | Satisfied | 29 | 5.6 | 5.6 | 99.0 |
|  | Very Satisfied | 5 | 1.0 | 1.0 | 100.0 |
|  | Total | 517 | 100.0 | 100.0 |  |

| **Table 20: Satisfaction with the distance to place of work from your house** | | | | | |
| --- | --- | --- | --- | --- | --- |
|  | | Frequency | Percent | Valid Percent | Cumulative Percent |
| Valid | No Response | 16 | 3.1 | 3.1 | 3.1 |
|  | Very Dissatisfied | 38 | 7.4 | 7.4 | 10.4 |
|  | Dissatisfied | 59 | 11.4 | 11.4 | 21.9 |
|  | Not Sure | 194 | 37.5 | 37.5 | 59.4 |
|  | Satisfied | 167 | 32.3 | 32.3 | 91.7 |
|  | Very Satisfied | 43 | 8.3 | 8.3 | 100.0 |
|  | Total | 517 | 100.0 | 100.0 |  |

| **Table 21: Satisfaction with access to healthcare facilities and service from your house** | | | | | |
| --- | --- | --- | --- | --- | --- |
|  | | Frequency | Percent | Valid Percent | Cumulative Percent |
| Valid | No Response | 8 | 1.5 | 1.5 | 1.5 |
|  | Very Dissatisfied | 185 | 35.8 | 35.8 | 37.3 |
|  | Dissatisfied | 172 | 33.3 | 33.3 | 70.6 |
|  | Not Sure | 112 | 21.7 | 21.7 | 92.3 |
|  | Satisfied | 31 | 6.0 | 6.0 | 98.3 |
|  | Very Satisfied | 9 | 1.7 | 1.7 | 100.0 |
|  | Total | 517 | 100.0 | 100.0 |  |

| **Table 22: Satisfaction with access to public infrastructure and urban services from your house** | | | | | |
| --- | --- | --- | --- | --- | --- |
|  | | Frequency | Percent | Valid Percent | Cumulative Percent |
| Valid | No Response | 4 | .8 | .8 | .8 |
|  | Very Dissatisfied | 58 | 11.2 | 11.2 | 12.0 |
|  | Dissatisfied | 157 | 30.4 | 30.4 | 42.4 |
|  | Not Sure | 231 | 44.7 | 44.7 | 87.0 |
|  | Satisfied | 61 | 11.8 | 11.8 | 98.8 |
|  | Very Satisfied | 6 | 1.2 | 1.2 | 100.0 |
|  | Total | 517 | 100.0 | 100.0 |  |

| **Table 23: Satisfaction with the prices of goods and services within and around the housing estate** | | | | | |
| --- | --- | --- | --- | --- | --- |
|  | | Frequency | Percent | Valid Percent | Cumulative Percent |
| Valid | No Response | 28 | 5.4 | 5.4 | 5.4 |
|  | Very Dissatisfied | 171 | 33.1 | 33.1 | 38.5 |
|  | Dissatisfied | 171 | 33.1 | 33.1 | 71.6 |
|  | Undecided | 123 | 23.8 | 23.8 | 95.4 |
|  | Satisfied | 19 | 3.7 | 3.7 | 99.0 |
|  | Very Satisfied | 5 | 1.0 | 1.0 | 100.0 |
|  | Total | 517 | 100.0 | 100.0 |  |

| **Table 24: Satisfaction with the extent to which the design of the residence suits your natural way of life** | | | | | |
| --- | --- | --- | --- | --- | --- |
|  | | Frequency | Percent | Valid Percent | Cumulative Percent |
| Valid | No Response | 4 | .8 | .8 | .8 |
|  | Very Dissatisfied | 6 | 1.2 | 1.2 | 1.9 |
|  | Dissatisfied | 48 | 9.3 | 9.3 | 11.2 |
|  | Not Sure | 316 | 61.1 | 61.1 | 72.3 |
|  | Satisfied | 118 | 22.8 | 22.8 | 95.2 |
|  | Very satisfied | 25 | 4.8 | 4.8 | 100.0 |
|  | Total | 517 | 100.0 | 100.0 |  |

| **Table 25: Satisfaction with residents rules and regulations in the housing estate** | | | | | |
| --- | --- | --- | --- | --- | --- |
|  | | Frequency | Percent | Valid Percent | Cumulative Percent |
| Valid | Response | 3 | .6 | .6 | .6 |
|  | Very Dissatisfied | 7 | 1.4 | 1.4 | 1.9 |
|  | Dissatisfied | 22 | 4.3 | 4.3 | 6.2 |
|  | Not Sure | 316 | 61.1 | 61.1 | 67.3 |
|  | Satisfied | 148 | 28.6 | 28.6 | 95.9 |
|  | Very Satisfied | 21 | 4.1 | 4.1 | 100.0 |
|  | Total | 517 | 100.0 | 100.0 |  |

| **Table 26: Satisfaction with existing management and maintenance practices in the housing estate** | | | | | |
| --- | --- | --- | --- | --- | --- |
|  | | Frequency | Percent | Valid Percent | Cumulative Percent |
| Valid | No Response | 5 | 1.0 | 1.0 | 1.0 |
|  | Very Dissatisfied | 45 | 8.7 | 8.7 | 9.7 |
|  | Dissatisfied | 238 | 46.0 | 46.0 | 55.7 |
|  | Not Sure | 146 | 28.2 | 28.2 | 83.9 |
|  | Satisfied | 64 | 12.4 | 12.4 | 96.3 |
|  | Very Satisfied | 19 | 3.7 | 3.7 | 100.0 |
|  | Total | 517 | 100.0 | 100.0 |  |

| **Table 27: Satisfaction with the level of communal activities in the housing estate** | | | | | |
| --- | --- | --- | --- | --- | --- |
|  | | Frequency | Percent | Valid Percent | Cumulative Percent |
| Valid | No Response | 14 | 2.7 | 2.7 | 2.7 |
|  | Very Dissatisfied | 79 | 15.3 | 15.3 | 18.0 |
|  | Dissatisfied | 54 | 10.4 | 10.4 | 28.4 |
|  | Not Sure | 277 | 53.6 | 53.6 | 82.0 |
|  | Satisfied | 78 | 15.1 | 15.1 | 97.1 |
|  | Very Satisfied | 15 | 2.9 | 2.9 | 100.0 |
|  | Total | 517 | 100.0 | 100.0 |  |

| **Table 28: Satisfaction with business and job opportunities within and around the housing estate** | | | | | |
| --- | --- | --- | --- | --- | --- |
|  | | Frequency | Percent | Valid Percent | Cumulative Percent |
| Valid | No Response | 36 | 7.0 | 7.0 | 7.0 |
|  | Very Dissatisfied | 142 | 27.5 | 27.5 | 34.4 |
|  | Dissatisfied | 159 | 30.8 | 30.8 | 65.2 |
|  | Undecided | 154 | 29.8 | 29.8 | 95.0 |
|  | Satisfied | 20 | 3.9 | 3.9 | 98.8 |
|  | Very Dissatisfied | 6 | 1.2 | 1.2 | 100.0 |
|  | Total | 517 | 100.0 | 100.0 |  |

| **Table 29: Satisfaction with the level of cleanliness of the housing estate** | | | | | |
| --- | --- | --- | --- | --- | --- |
|  | | Frequency | Percent | Valid Percent | Cumulative Percent |
| Valid | No Response | 2 | .4 | .4 | .4 |
|  | Very Dissatisfied | 11 | 2.1 | 2.1 | 2.5 |
|  | Dissatisfied | 157 | 30.4 | 30.4 | 32.9 |
|  | Undecided | 234 | 45.3 | 45.3 | 78.1 |
|  | Satisfied | 93 | 18.0 | 18.0 | 96.1 |
|  | Very Satisfied | 20 | 3.9 | 3.9 | 100.0 |
|  | Total | 517 | 100.0 | 100.0 |  |

| **Table 30: Satisfaction with the level of crime and anti-social activities in the housing estate** | | | | | |
| --- | --- | --- | --- | --- | --- |
|  | | Frequency | Percent | Valid Percent | Cumulative Percent |
| Valid | No Response | 5 | 1.0 | 1.0 | 1.0 |
|  | Very Dissatisfied | 10 | 1.9 | 1.9 | 2.9 |
|  | Dissatisfied | 22 | 4.3 | 4.3 | 7.2 |
|  | Not Sure | 245 | 47.4 | 47.4 | 54.5 |
|  | Satisfied | 199 | 38.5 | 38.5 | 93.0 |
|  | Very Satisfied | 36 | 7.0 | 7.0 | 100.0 |
|  | Total | 517 | 100.0 | 100.0 |  |

| **Table 31: Satisfaction with the security of life and property in the housing estate** | | | | | |
| --- | --- | --- | --- | --- | --- |
|  | | Frequency | Percent | Valid Percent | Cumulative Percent |
| Valid | No Response | 1 | .2 | .2 | .2 |
|  | Very Dissatisfied | 15 | 2.9 | 2.9 | 3.1 |
|  | Dissatisfied | 12 | 2.3 | 2.3 | 5.4 |
|  | Undecided | 280 | 54.2 | 54.2 | 59.6 |
|  | Satisfied | 178 | 34.4 | 34.4 | 94.0 |
|  | Very Satisfied | 31 | 6.0 | 6.0 | 100.0 |
|  | Total | 517 | 100.0 | 100.0 |  |

| **Table 32: Descriptive Statistics of Residents satisfaction with different components of their residential environment** | | | | |
| --- | --- | --- | --- | --- |
|  | N | Mean | Std. Deviation | Rank |
| Satisfaction with the level of privacy in the dwelling unit | 517 | 3.8936 | .82023 | 1 |
| Satisfaction with size of bedrooms in the housing unit | 517 | 3.7950 | .77793 | 2 |
| Satisfaction with the sizes of living/dining space | 517 | 3.6460 | .84476 | 3 |
| Satisfaction with the level of noise pollution within the estate | 517 | 3.4487 | .77496 | 4 |
| Satisfaction with the location of housing unit within the estate | 517 | 3.4275 | .80518 | 5 |
| Satisfaction with the level of crime and anti-social activities in the housing estate | 517 | 3.4139 | .83691 | 6 |
| Satisfaction with the sizes of cooking and storage spaces | 517 | 3.3888 | 1.05549 | 7 |
| Satisfaction with the housing type | 517 | 3.3888 | .76611 | 8 |
| Satisfaction with the security of life and property in the housing estate | 517 | 3.3772 | .77441 | 9 |
| Satisfaction with the cost of acquisition or rent paid for the residence | 517 | 3.3675 | .88722 | 10 |
| Satisfaction with natural lighting and ventilation of living and sleeping areas in the house | 517 | 3.3308 | .67405 | 11 |
| Satisfaction with residents rules and regulations in the housing estate | 517 | 3.2805 | .72113 | 12 |
| Satisfaction with the aesthetics of the house | 517 | 3.2495 | .82689 | 13 |
| Satisfaction with bathroom and toilet facilities in the dwelling unit | 517 | 3.2263 | .87882 | 14 |
| Satisfaction with the extent to which the design of the residence suits your natural way of life | 517 | 3.1857 | .77281 | 15 |
| Satisfaction with the distance to place of work from your house | 517 | 3.1354 | 1.15178 | 16 |
| Satisfaction with the building materials used | 517 | 3.0213 | .89439 | 17 |
| Satisfaction with the number of bedrooms in the dwelling units | 517 | 3.0077 | 1.05835 | 18 |
| Satisfaction with the level of cleanliness of the housing estate | 517 | 2.8994 | .86577 | 19 |
| Satisfaction with the level of communal activities in the housing estate | 517 | 2.7176 | 1.07181 | 20 |
| Satisfaction with access to public infrastructure and urban services from your house | 517 | 2.5899 | .90548 | 21 |
| Satisfaction with existing management and maintenance practices in the housing estate | 517 | 2.5338 | .97513 | 22 |
| Satisfaction with power supply in the residence | 517 | 2.4584 | .91748 | 23 |
| Satisfaction with water supply in the dwelling unit | 517 | 2.4429 | .88217 | 24 |
| Satisfaction with the distance to children's school from your house | 517 | 2.4062 | 1.13327 | 25 |
| Satisfaction with the distance from your house to the nearest market | 517 | 2.1238 | 1.06027 | 26 |
| Satisfaction with access to healthcare facilities and service from your house | 517 | 2.0000 | 1.01729 | 27 |
| Satisfaction with business and job opportunities within and around the housing estate | 517 | 1.9961 | 1.05470 | 28 |
| Satisfaction with the distance from your house to recreational facilities | 517 | 1.9014 | .87600 | 29 |
| Satisfaction with the prices of goods and services within and around the housing estate | 517 | 1.9014 | 1.00577 | 30 |
| Satisfaction with distance between your house and nearest shopping facilities | 517 | 1.8627 | .96172 | 31 |
