# Supplementary material for: Survey dataset on occupants׳ satisfaction with housing and life in public residential estates in Ogun State, Nigeria
Source: Data Brief. 2018 Sep 15;20:1745–8. doi: 10.1016/j.dib.2018.09.036 (PMC6161412; doi:10.1016/j.dib.2018.09.036)
Supplement: Supplementary file 2 — Supplementary material [file mmc2.zip › Housing units' Characteristics.doc]

| **Number of Bedrooms in the dwelling units** | | | | | |
| --- | --- | --- | --- | --- | --- |
|  | | Frequency | Percent | Valid Percent | Cumulative Percent |
| Valid | 1 B/Rm | 88 | 17.0 | 17.0 | 17.0 |
|  | 2 B/Rms | 184 | 35.6 | 35.6 | 52.6 |
|  | 3 B/Rms | 219 | 42.4 | 42.4 | 95.0 |
|  | 4B/Rms | 22 | 4.3 | 4.3 | 99.2 |
|  | More Than 5B/Rms | 4 | .8 | .8 | 100.0 |
|  | Total | 517 | 100.0 | 100.0 |  |

| **Residence type** | | | | | |
| --- | --- | --- | --- | --- | --- |
|  | | Frequency | Percent | Valid Percent | Cumulative Percent |
| Valid | Single family Bungalow | 266 | 51.5 | 51.5 | 51.5 |
|  | Semi-Detached Bugalow | 238 | 46.0 | 46.0 | 97.5 |
|  | Duplex | 13 | 2.5 | 2.5 | 100.0 |
|  | Total | 517 | 100.0 | 100.0 |  |

| **Location of Kitchen in the House** | | | | | |
| --- | --- | --- | --- | --- | --- |
|  | | Frequency | Percent | Valid Percent | Cumulative Percent |
| Valid | Within the Building | 517 | 100.0 | 100.0 | 100.0 |

| **Use of Kitchen** | | | | | |
| --- | --- | --- | --- | --- | --- |
|  | | Frequency | Percent | Valid Percent | Cumulative Percent |
| Valid | Exclusive for a Family | 517 | 100.0 | 100.0 | 100.0 |

| **Modes of water supply in the residence** | | | | | |
| --- | --- | --- | --- | --- | --- |
|  | | Frequency | Percent | Valid Percent | Cumulative Percent |
| Valid | No Response | 1 | .2 | .2 | .2 |
|  | Water Vendors | 48 | 9.3 | 9.3 | 9.5 |
|  | Wells | 72 | 13.9 | 13.9 | 23.4 |
|  | Boreholes within the Estate | 251 | 48.5 | 48.5 | 72.0 |
|  | Public Water Supply System | 5 | 1.0 | 1.0 | 72.9 |
|  | Private Borehole | 121 | 23.4 | 23.4 | 96.3 |
|  | Water Vendors and Wells | 13 | 2.5 | 2.5 | 98.8 |
|  | Water vendors and Private Borehole | 4 | .8 | .8 | 99.6 |
|  | Water vendors and Borehole in the Estate | 2 | .4 | .4 | 100.0 |
|  | Total | 517 | 100.0 | 100.0 |  |

| **Main source of power supply in the dwelling units** | | | | | |
| --- | --- | --- | --- | --- | --- |
|  | | Frequency | Percent | Valid Percent | Cumulative Percent |
| Valid | No Response | 1 | .2 | .2 | .2 |
|  | Personal Power Generaing Set | 48 | 9.3 | 9.3 | 9.5 |
|  | Housing Estate Power generating Plant(s) | 2 | .4 | .4 | 9.9 |
|  | Public Power Supply | 254 | 49.1 | 49.1 | 59.0 |
|  | Solar Panel | 2 | .4 | .4 | 59.4 |
|  | Public Power Supply and Personal Generator | 210 | 40.6 | 40.6 | 100.0 |
|  | Total | 517 | 100.0 | 100.0 |  |

| **Refuse disposal arrangement** | | | | | |
| --- | --- | --- | --- | --- | --- |
|  | | Frequency | Percent | Valid Percent | Cumulative Percent |
| Valid | No Response | 5 | 1.0 | 1.0 | 1.0 |
|  | Residents | 322 | 62.3 | 62.3 | 63.2 |
|  | Contractors | 96 | 18.6 | 18.6 | 81.8 |
|  | The Estate Managers | 31 | 6.0 | 6.0 | 87.8 |
|  | Government Agencies | 63 | 12.2 | 12.2 | 100.0 |
|  | Total | 517 | 100.0 | 100.0 |  |

| **Mode of acquisition of residence** | | | | | |
| --- | --- | --- | --- | --- | --- |
|  | | Frequency | Percent | Valid Percent | Cumulative Percent |
| Valid | No Response | 181 | 35.0 | 35.0 | 35.0 |
|  | Bought directly from the developers | 88 | 17.0 | 17.0 | 52.0 |
|  | Bought from previous owner(s) | 12 | 2.3 | 2.3 | 54.4 |
|  | Government allocation | 108 | 20.9 | 20.9 | 75.2 |
|  | Inherited | 111 | 21.5 | 21.5 | 96.7 |
|  | Mortgage Arrangement | 1 | .2 | .2 | 96.9 |
|  | Gift | 16 | 3.1 | 3.1 | 100.0 |
|  | Total | 517 | 100.0 | 100.0 |  |

| **Walling materials used** | | | | | |
| --- | --- | --- | --- | --- | --- |
|  | | Frequency | Percent | Valid Percent | Cumulative Percent |
| Valid | Sun dried burnt bricks | 19 | 3.7 | 3.7 | 3.7 |
|  | Sancrete Cement Blocks | 498 | 96.3 | 96.3 | 100.0 |
|  | Total | 517 | 100.0 | 100.0 |  |

| **Wall finishes** | | | | | |
| --- | --- | --- | --- | --- | --- |
|  | | Frequency | Percent | Valid Percent | Cumulative Percent |
| Valid | Paint | 508 | 98.3 | 98.3 | 98.3 |
|  | Facing bricks | 9 | 1.7 | 1.7 | 100.0 |
|  | Total | 517 | 100.0 | 100.0 |  |

| **Window types** | | | | | |
| --- | --- | --- | --- | --- | --- |
|  | | Frequency | Percent | Valid Percent | Cumulative Percent |
| Valid | Glazed Louvre | 103 | 19.9 | 19.9 | 19.9 |
|  | Glazed aluminium | 414 | 80.1 | 80.1 | 100.0 |
|  | Total | 517 | 100.0 | 100.0 |  |

| **Door types** | | | | | |
| --- | --- | --- | --- | --- | --- |
|  | | Frequency | Percent | Valid Percent | Cumulative Percent |
| Valid | Plywood Flushed | 2 | .4 | .4 | .4 |
|  | Pannelled Timber | 207 | 40.0 | 40.0 | 40.4 |
|  | Aluminium Glazed | 3 | .6 | .6 | 41.0 |
|  | Panelled Steel | 305 | 59.0 | 59.0 | 100.0 |
|  | Total | 517 | 100.0 | 100.0 |  |

| **Floor finish** | | | | | |
| --- | --- | --- | --- | --- | --- |
|  | | Frequency | Percent | Valid Percent | Cumulative Percent |
| Valid | Cement Screed | 338 | 65.4 | 65.4 | 65.4 |
|  | Ceramic Tiles | 179 | 34.6 | 34.6 | 100.0 |
|  | Total | 517 | 100.0 | 100.0 |  |

| **Ceiling materials** | | | | | |
| --- | --- | --- | --- | --- | --- |
|  | | Frequency | Percent | Valid Percent | Cumulative Percent |
| Valid | Asbestos | 480 | 92.8 | 92.8 | 92.8 |
|  | Mineral fiber | 22 | 4.3 | 4.3 | 97.1 |
|  | Acoustic Ceiling | 4 | .8 | .8 | 97.9 |
|  | PVC Strips | 11 | 2.1 | 2.1 | 100.0 |
|  | Total | 517 | 100.0 | 100.0 |  |

| **Type of roofing sheets used** | | | | | |
| --- | --- | --- | --- | --- | --- |
|  | | Frequency | Percent | Valid Percent | Cumulative Percent |
| Valid | Aluminium Long Span | 502 | 97.1 | 97.1 | 97.1 |
|  | Villa Tiles | 15 | 2.9 | 2.9 | 100.0 |
|  | Total | 517 | 100.0 | 100.0 |  |

| **State of repair of residence** | | | | | |
| --- | --- | --- | --- | --- | --- |
|  | | Frequency | Percent | Valid Percent | Cumulative Percent |
| Valid | Minor Repairs | 36 | 7.0 | 7.0 | 7.0 |
|  | Sound | 481 | 93.0 | 93.0 | 100.0 |
|  | Total | 517 | 100.0 | 100.0 |  |
